# HIV Replication Enhances Production of Free Fatty Acids, Low Density Lipoproteins and Many Key Proteins Involved in Lipid Metabolism: A Proteomics Study

**DOI:** 10.1371/journal.pone.0003003

**Published:** 2008-08-20

**Authors:** Suraiya Rasheed, Jasper S. Yan, Alex Lau, Arvan S. Chan

**Affiliations:** Laboratory of Viral Oncology, AIDS and Proteomics Research, Keck School of Medicine, University of Southern California, Los Angeles, California, United States of America; University of California San Francisco, United States of America

## Abstract

**Background:**

HIV-infected patients develop multiple metabolic abnormalities including insulin resistance, lipodystrophy and dyslipidemia. Although progression of these disorders has been associated with the use of various protease inhibitors and other antiretroviral drugs, HIV-infected individuals who have not received these treatments also develop lipid abnormalities albeit to a lesser extent. How HIV alters lipid metabolism in an infected cell and what molecular changes are affected through protein interaction pathways are not well-understood.

**Results:**

Since many genetic, epigenetic, dietary and other factors influence lipid metabolism *in vivo*, we have chosen to study genome-wide changes in the proteomes of a human T-cell line before and after HIV infection in order to circumvent computational problems associated with multiple variables. Four separate experiments were conducted including one that compared 14 different time points over a period of >3 months. By subtractive analyses of protein profiles overtime, several hundred differentially expressed proteins were identified in HIV-infected cells by mass spectrometry and each protein was scrutinized for its biological functions by using various bioinformatics programs. Herein, we report 18 HIV-modulated proteins and their interaction pathways that enhance fatty acid synthesis, increase low density lipoproteins (triglycerides), dysregulate lipid transport, oxidize lipids, and alter cellular lipid metabolism.

**Conclusions:**

We conclude that HIV replication alone (i.e. without any influence of antiviral drugs, or other human genetic factors), can induce novel cellular enzymes and proteins that are significantly associated with biologically relevant processes involved in lipid synthesis, transport and metabolism (p = <0.0002–0.01). Translational and clinical studies on the newly discovered proteins may now shed light on how some of these proteins may be useful for early diagnosis of individuals who might be at high risk for developing lipid-related disorders. The target proteins could then be used for future studies in the development of inhibitors for preventing lipid-metabolic anomalies. This is the first direct evidence that HIV-modulates production of proteins that are significantly involved in disrupting the normal lipid-metabolic pathways.

## Introduction

In the past two decades, the use of highly active antiretroviral therapy (HAART) has minimized the global incidence of the acquired immunodeficiency syndrome (AIDS) by reducing the viral load in HIV-infected individuals [1]–[4]. However, a significant number of patients on HAART develop multiple metabolic complications and are at high risk for developing many chronic diseases including insulin resistance, coronary artery disease, renal diseases, lipodystrophy and dyslipidemia to name a few [1], [2], [5]–[14]. Although lipodystrophy is a rare disease in the general population, it is significantly common in HIV-infected individuals who have been treated with various combination therapies [14]–[21]. In addition, many HIV-infected individuals who have *not* received any treatment also develop hyperlipidemia, hypertension, diabetes, insulin resistance, and other metabolic complications [10], [16], [21].

Lipodystrophy was initially characterized as the wasting syndrome with selective loss of body fat or lipoatrophy (thinning of arms, legs and face) central adiposity and accumulation of fat in the breast tissue or the back of the neck (buffalo hump) [22], [23]. The clinical profile of this disease has now been expanded to include dyslipidemia, hyperglycemia, hypercholesterolemia, hepatomegaly, glomerulonephritis and other metabolic syndromes [4], [6], [15], [18], [20], [22], [24]–[26]. Once the symptomatic disease is established in HIV-infected patients, it is difficult to reverse the condition in these individuals [27].

Although molecular mechanisms responsible for the numerous lipid-related disorders in HIV-infected individuals are not well understood , treatment with nucleoside reverse transcriptase inhibitors (NRTI's) has been reported to affect mitochondrial functions by depletion of its DNA and inhibiting transcription [27]. On the other hand, protease inhibitors (PI's) bind to catalytic domains of the HIV protease and inhibit virus replication [4], [18], [23], [27], [28]. PI's also bind to the low-density lipoprotein-receptor-related protein (LRP) and cytoplasmic retinoic-acid binding protein type 1 (CRABP1), both of which show 60% sequence homologies to HIV protease [18]. Since these proteins regulate lipid metabolism, the binding of PI's to LRP and CRABP1 impairs chylomicron uptake and triglyceride clearance [18], [23]. In addition, adipocyte toxicity has also been reported due to PI's interference with the functions of the transcription factor and the sterol regulatory element binding protein 1C [27].

Among the numerous cell types that are susceptible to HIV infection *in vivo*, the T-cells, monocytes, macrophages, follicular dendritic cells are primarily responsible for the enhanced replication of the virus in the body. Although adipocytes can be infected by HIV *in vitro* or *in vivo*, these cells are not uniformly infected and the virus replication is slow because most adipocytes are quiescent cells. Exposure of these cells to cytokines or other factors produced by a variety of cell types infected by HIV (or other pathogenic organisms) activates expression of HIV receptors needed for the infection and replication [29]–[32]. Further, adipose tissue is comprised of multiple cell types including adipocytes, monocytes, macrophages, endothelial and vascular smooth muscle cells [30], [32], [33]. These immune cells are functionally active in the adipose tissues and produce numerous cytokines and other regulatory factors that influence endocrine and lipid metabolism [32], [33]. The adipokines are also vital for lipid homeostasis, regulation of steroid hormones, prostaglandin and fat soluble vitamins [30], [34], [35]. These factors also control storage of excess lipids and triglycerides (both normal and abnormal fatty acids) present in the circulation [34], [36]. Many infectious agents including HIV have profound effects on adipocytes which become dysfunctional and can not store most lipids /triglycerides properly. This causes additional lipid abnormalities and metabolic disorders in HIV-infected individuals [20], [30], [37]. Therefore, it is important to note that even when adipocytes are infected by HIV *in vivo,* the multiple abnormalities seen in adipose tissues of HIV-1-infected individuals are not due to the *replication* of HIV in adipocytes *per se*.

The viral envelope and cell surface membranes are made up of lipoproteins and cholesterols, and the fatty acids are synthesized in the cytoplasm. Both lipids and lipoproteins are required for molecular communication at each step of the virus replication, from its entry into the cell to integration, transcription, assembly and budding of virus particles from cell membranes [14], [38]–[44]. While the bulk of lipids present in the circulation are contributed by the dietary fats, at least eight different types of lipids are synthesized in various cell types [30], [33], [37]. The lipid-binding acyl proteins are catalyzed by a number of enzymes to make different types and amounts of lipids that are required for the construction of cellular membranes. Further, cholesterol efflux is a common phenomenon in HIV-infected lymphocytes and macrophages [45]–[48].When excess lipids are synthesized they are leached out from various cell types (in circulation) and are stored in the adipocytes, preadipocytes , undifferentiated fibroblasts or mesenchymal connective tissue cells that are present in many organs [30], [32].

Recently several investigators have used RNA expression profiles, single nucleotide polymorphisms (SNP's) and proteomics technology to understand the inner workings of HIV-infected and uninfected cells. Whereas gene expression data provides vital genetic information [34], [49]–[51], the mRNAs are modified post-transcriptionally and their products are altered post-translationally to give rise to disease-specific proteins. While a number of viral and cellular proteins involved in cell cycle, HIV-induced syncytia formation, apoptosis, and cytopathicity of infected macrophages have been identified by proteomics technology [52]–[57], molecular events involved in HIV-related lipid abnormalities have not yet been studied. Since the cellular systems and cellular milieu of proteins help in the synthesis, distribution and maintenance of lipid homeostasis within various tissues [30], [35], we chose to study proteins involved in lipid metabolism by HIV infection alone without any influence of the genetic diversity of HIV and the human population it infects. A major consideration in the design of our experiments was to circumvent numerous computational problems associated with genetic and epigenetic variables (smoking, alcohol, fat-rich diet, diabetes, blood pressure, diabetes etc.) that influence lipid metabolism *in vivo*. Thus, we also avoided testing proteomes of mononuclear cells derived from freshly collected peripheral blood because the susceptibility of these genetically diverse cells to HIV infection would be different and the significance of variables would be difficult to assess in a heterogeneous human population. Likewise, we did not use primary HIV strains that are uniquely distinct as a quasispecies in each HIV-infected individual. In addition, HIV replication as well as disease outcomes are influenced by TNF and other cytokines produced by many pathogenic viruses such as hepatitis, herpes and other microbes that co-inhabit HIV infected individuals. Since efficient replication of the virus is essential to the disease development, we chose a single-cell clone of a human T-cell line (RH9) that is highly susceptible to HIV infection and studied changes in protein profiles due to infection with a biologically cloned HIV strain B (X4) *in vitro* [58], [59]. Cellular proteins were analyzed by proteomics technology (two-dimensional gel electrophoresis (2DGE), image analyses, mass spectrometry, bioinformatics and statistical analyses. Multiple sets of proteomes were evaluated over a 3-year period including one experiment which compared 13–14 different time points (starting at 1.5 hrs to 96 days post HIV-infection) over three months (see materials and methods).

In this report we present the first direct evidence that HIV replication alone in human T-cells, without any influence of antiviral drugs or other factors, can stimulate production of novel cellular enzymes and proteins that enhance fatty acid synthesis, increase quantity of low density lipoproteins, secrete triglycerides, dysregulate lipid transport, oxidize lipids, and alter lipid metabolism. This data leads us to a new concept in HIV-induced disease mechanisms.

## Results and Discussion

### 1. Identification of Differentially Regulated Proteins Post-HIV Infection

**Figure 1:**
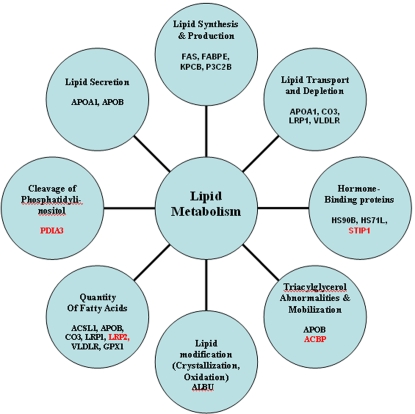
Diagram showing putative biological processes involved in lipid metabolism. Each circle represents proteins associated with the respective function/s. Proteins in red were downregulated post-HIV infection (Figure 3). All other proteins were either expressed exclusively in HIV infected cells (Figure 2) or upregulated after virus infection (Figure 3). Full names, abbreviations and accession numbers of each protein are listed in Tables 1&2.

We have identified 18 proteins that have been differentially regulated after HIV infection *in vitro* (Figure 1 & Tables 1&2). Stringent protocols were used to select lipid associated proteins from several hundred proteins that were upregulated, downregulated, or synthesized *de novo* in HIV-infected cells. All proteins were identified by Matrix Assisted Laser Desorption Time Of Flight (MALDI- TOF) mass spectrometry (MS) and confirmed from multiple gels. Biological functions of each protein were scrutinized by at least four investigators from the global public databases and by the use of several bioinformatics programs. The Ingenuity Systems' knowledgebase of the “Functional Repository of Human Genes and Proteins” was used to confirm proteins involved in different aspects of human lipid metabolism. The Ingenuity System was also used to calculate the likelihood of whether a specific lipid-related function in the “Global Functional Analysis” and “Global Canonical Pathways” is due to random chance or the given biological process in lipid metabolic pathway is statistically significant. Only those proteins that showed a p-value of <0.05 in relation to their effects on lipid metabolism were selected for further analyses. Each of the selected proteins could be categorically associated with one or more aspects of the lipid metabolism including the synthesis, production, secretion and transport of various fatty acids (Figure 1).

**Table 1:** HIV- Modulated Enzymes and Kinases Associated with Fatty Acid Synthesis and Lipid Metabolism.

Protein Name	Abbrev.	Acces. #	Putative Function	P-value	Location
Long-chain-fatty-acid-CoA ligase 1 (synonym: Acyl-CoA synthetase 1)	ACSL1	P41215	Provides activated intermediates for complex lipid synthesis, protein modification, and beta-oxidation	0.005 to 0.02	mitochondria/endoplasmic reticulum/peroxisome/microsomal membrane
Complement C3 precursor	CO3 (C3)	P01024	Peptidase that promotes virus infection and fatty acid synthesis; cooperates with other lipogenic proteins in removal of triaclglycerols	0.0002 to 0.005	extra-cellular space
Fatty acid synthase	FAS (FASN)	P49327	Lipogenic enzyme:catalyzes formation of long-chain fatty acids	0.02	cytoplasm, melanosome
Glutathione peroxidase	GPX1	P07203	Antioxidant enzyme that detoxifies lipid hydroperoxides	0.01	cytoplasm
Protein kinase C beta type	KPCB (PRKCB1)	P05771	Is involved in generation of phosphatidylethanolamine, a lipid found in biological membranes	0.01	cytoplasm, peripheral membrane
Phosphatidylinositol-4-phosphate 3-kinase C2 domain-containing beta polypeptide	P3C2B (PIK3C2B)	O00750	Lipid kinase activity– phosphorylates phosphatidylinositol in the cell membrane	0.02	plasma membrame, cytoplasm, microsome, nucleus
Protein disulfide isomerase A3 precursor	PDIA3*	P30101	Essential for the formation of APOB;catalyzes rearrangement of -S-S- bonds in proteins	0.02	endoplasmic reticulum lumen, melanosome

All proteins except PDIA3 (^*^) were upregulated compared to the counterpart uninfected cells or were synthesized de novo in HIV-infected cells. Protein names, abbreviations, locations and accession numbers are from SwissProt and are presented in alphabetical order. Putative functions of each protein are related to biological processes involved in various aspects of lipid metabolism. Significance associated with lipid metabolism was calculated by Fisher Exact Test using software from Ingenuity Systems Inc. A p-value of <0.05 represents a statistically significant non-random association of a specific protein with a lipid-related function in the Global Functional Analysis.

**Table 2:** HIV- Modulated Proteins Associated with Lipid Metabolism

Protein Name	Abbrev.	Acces. #	Putative Function	P-value	Location
Acyl-CoA-binding protein	ACBP*	P07108	Loss or deficiency of ACBP results in fatty acid metabolism abnormalities	0.02	cytoplasm; Ligand
Serum albumin [Precursor]	ALBU (ALB)	P02768	Binds to fatty acids: lipid peroxidation and crystallization	0.01	Extra-cellular space; Transporter
Apolipoprotein A-I	APOA1	P02647	Involved in reverse transport of cholesterol from tissues; promotes cholesterol efflux from tissues; related to ipoprotein-modifying enzymes degrades APOB	0.03	Extra-cellular space;Transporter
Apolipoprotein B-100 [Precursor]	APOB	P04114	Major protein constituent of LDL and VLDL. Functions as a recognition signal for the cellular binding and internalization of LDL particles by the apoB/E receptor; only protein to mobilize triacylglycerides	0.03	Extra-cellular space;Transporter
Fatty acid-binding protein, epidermal	FABPE (FABP5)	Q01469	High specificity for fatty acids. Involved in biosynthesis of phosphatidylcholine and other lipids	0.02	Adapter/Transporter
Heat shock 70 kDa protein 1-HOM	HS71L (HSPA1L)	P34931	Functions in protein folding; prolongs life of mature protein kinase C by allowing the enzyme to re-phosphorylate	0.0002	cytoplasm; Stress-induced Molecular Chaperone
Heat shock protein HSP 90-beta	HS90B (HSP90AB1)	P08238	molecular chaperone works with HS71L	0.0002	cytoplasm, melanosome; Stress-induced Molecular Chaperone
Low-density lipoprotein receptor-related protein 1 precursor	LRP1	Q07954	Receptor for aggregated LDL ; increases quantity of free fatty acids	0.0002 to 0.002	plasma membrane, cytoplasm, nucleus; Receptor
Low-density lipoprotein receptor-related protein 2 precursor	LRP2*	P98164	Regulates lipid metabolism, modulates multiple signaling pathways	0.02	membrane; Receptor
Stress-induced-phosphoprotein 1	STIP1*	P31948	Mediates the association of the molecular chaperones HSC70 and HSP90; downregulation may enhance lipid oxidation	0.0002	cytoplasm, nucleus;Adapter
Very low-density lipoprotein receptor precursor	VLDLR	P98155	Increases quantities of LDL and fatty acids; reduces clearance of triacylglycerol-rich lipids	0.0002 to 0.01	membrane; Receptor/Transporter

The three downregulated proteins are marked with (^*^). All other (n = 8) proteins were either expressed exclusively in HIV-infected cells or were upregulated compared to those detected in the uninfected cells. Protein names, abbreviations, locations and accession numbers are according to SwissProt. Putative functions and significance scores in relation to lipid metabolism were calculated by Fisher Exact Test using software from Ingenuity Systems Inc. A p-value of <0.05 represents a statistically significant non-random association of a specific protein with a lipid-related function/s in the Global Functional Analysis

**Figure 2:**
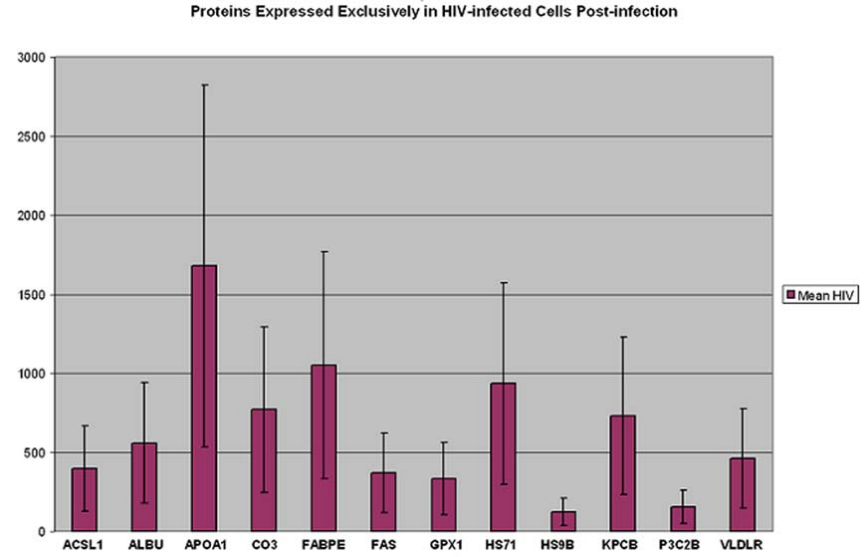
Proteins detected exclusively in HIV-infected cells by MALDI-TOF mass spectrometry from multiple gels. These proteins were not detected in counterpart uninfected cells tested at multiple time points and various stages of cell growth. X-axis = protein names (abbreviations) are according to SwissPROT: Y-axis = average of normalized quantity and standard deviations for each protein expressed in multiple gels. The line limits are +/− one standard deviation for the range of data points for each protein. Full protein names and Accession #s of each protein are provided in Table 1.

**Figure 3:**
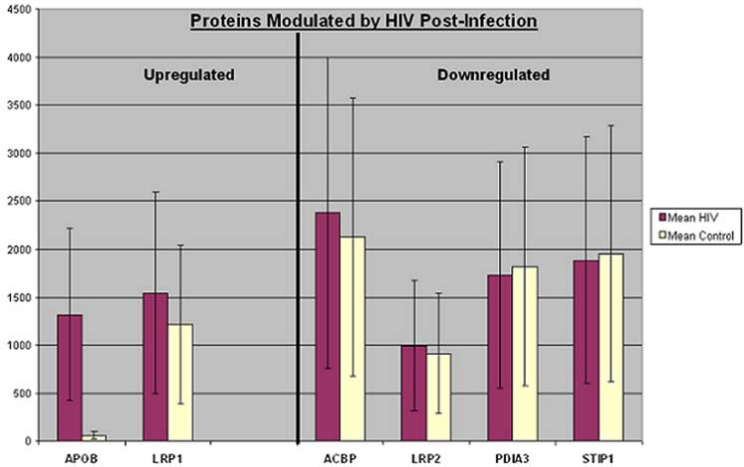
Proteins modulated by HIV post-infection. Up-regulated proteins are APOB and LRP1 and downregulated proteins are ACBP, STIP1, LRP2, and PDIA3. X-axis = protein names (abbreviations according to SwissPROT). Each protein was detected in multiple gels. Y-Axis = average of normalized quantities and standard deviations for each protein detected in multiple gels. The line limits are +/− one standard deviation for the range of data points for each protein. Full protein names and Accession #s of each protein are provided in Tables 1 & 2.

More than 66% (12 of 18) of the identified proteins were detected exclusively in HIV-infected cells (i.e. produced *de novo* after HIV infection). These proteins were not detected in the counterpart uninfected cells tested at different phases of cell growth or at each of the 14 time points over a period of >3 months (Figures 2). Two of the existing proteins were upregulated and four proteins were downregulated post-infection (Figure 3).

A functional categorization of each protein indicated that seven proteins belonged to various families of enzymes/kinases; five were transporter proteins, two transmembrane receptors, two molecular chaperones, and one each of the ligand-binding and adapter-like proteins (Tables 1& 2). The precursors of most of the secretory proteins could be located in the cytoplasm or cytoskeleton.

Herein, we demonstrate that each of the differentially regulated proteins (scripted in bold), is biologically relevant and has been shown to play statistically significant role/s in cellular functions associated with lipid metabolism.

### 2. Enzymes and Proteins Involved in Enhanced Fatty Acid Synthesis

One of the most essential biological processes involved in dyslipidemia and lipodystrophy syndrome is the accumulation of lipids and disproportionate distribution of *tissue-associated* fats due to the enhanced fatty acid synthesis. Since kinases and enzymes activate most cellular functions including lipid synthesis, we first analyzed the functional significance of these proteins in HIV-infected cells in comparison with those expressed in the uninfected control cells.

Of the 18 differentially expressed proteins in HIV-infected cells, six enzymes/kinases were expressed exclusively in HIV-infected cells (CO3, P3C2B, KPCB, FAS, ACSL1, and GPX1) and one isomerase (PDIA3) was slightly downregulated after chronic HIV infection (Table 1& Figure 3). All of these enzymes/ peptidase/kinases have been shown to be essential for the fatty acid synthesis as well as for numerous molecular interactions necessary for lipid metabolism (Table 1). None of these enzymes was detected in any of the corresponding gel-spots tested from the uninfected cells over a long period of growth (Table 1 & Figure 2).

**The complement C3 peptidase, or CO3 protein** was expressed exclusively in HIV-infected cells. This extracellular membrane bound peptidase was not detected in any of the numerous uninfected counterpart cells tested (Figure 2). The complement cascade is probably activated by HIV infection since C3- binding domains are present on HIV envelope gp120 as well as gp41 sequences [60]. This increases the rate of infection in different cell types of the body and contributes to HIV pathogenesis [61], [62]. Another important characteristic of the CO3 interaction with HIV is due to the sequence identity of the C3- activation peptide to an acylation-stimulating lipogenic protein produced by adipocytes [36], [63]. Since C3 is a primary contact point with HIV envelope and other cell surface proteins, the lipogenic process is most likely initiated very early in HIV infected cells (SR unpublished data). During lipogenesis, glucose is converted to fatty acids, which are subsequently esterified to glycerol to form triacylglycerols [64]. In the rat model system, elevated levels of CO3 were shown to be critical for *de novo* lipogenesis and these have been associated with liver lipodystrophy observed during myo-inositol deficiency [65], [66]. It is also noteworthy that high levels of CO3 protein in *non-HIV infected* individuals have been associated with higher lipid production, high blood pressure and risk of cardiovascular disease [67], [68]. Our functional bioinformatics analyses identified CO3 protein to be significantly associated with lipid-related proteins and therefore may increase the quantity of free fatty acids (p = 0.005). Additionally, CO3 is also involved in the removal and clearance of triacylglycerol (p = 0.0002) (Table 1& Figures 1& 2).

While the HIV-infected cells showed *de novo* expression of **phosphatidylinositol-4-phosphate 3-kinase C2** **domain-containing beta polypeptide** (**P3C2B or PI3K,** *)* at several time points, the uninfected T-cells did not (Figure 2 & Table 1). This membrane-bound enzyme is a major lipid messenger that may be activated soon after binding of the viral envelope to the plasma membrane [69]. In the initial phases of virus replication this kinase is involved in both receptor-mediated responses and intracellular signaling that are critical for cell survival and virus spread [69], [70]. This enzyme has been localized to the cytoplasmic lipid bodies, which mediate signals in lymphocytes and monocytes [71]. Furthermore, the expression of PI3K in mice with knockout phosphatidylinositol 5-phosphate 4-kinase (beta) gene was correlated with hypersensitivity to insulin [72]. These animals showed significantly less body fat compared to the wild-type mice indicating that PI3 kinase is essential for the lipid synthesis [72]. It is also notable that Nelfinavir, a protease inhibitor, can induce a defect in the insulin signaling cascade *downstream* of the activation of PI3-kinase *in vitro* [17]. These results suggest that HIV- induced enzymes can be further modulated by antiviral treatments. Our bioinformatics analysis indicated that the signaling activities of PI3K /P3C2B kinase are essential for lipid synthesis and adiposity (p = 0.02).

One of the most critical enzymes for the synthesis of fatty acids is the **fatty acid synthase** **(FAS** ***)***. This enzyme was detected only in HIV-infected cells and was not expressed in the uninfected counterpart T-cells (Figure 2). Recently another proteomics study reported upregulation of FAS after HIV infection and associated this enzyme to the dysregulation of the cell cycle post-HIV infection [56]. FAS has multiple domains of interactions that catalyze the formation of long-chain fatty acids and is therefore a key lipogenic enzyme in catalyzing the terminal steps in the *de novo* biogenesis of fatty acids [65], [66]. An increase of FAS has been associated with liver lipodystrophy and production of this enzyme was suppressed by polyunsaturated fatty acids, signifying its role in lipid synthesis and production of fatty acids [65], [66]. Our bioinformatics analysis also indicated that FAS is significantly associated with *de novo* lipogenesis and enhances the synthesis of myristate and stearic acids (p = 0.02).

A multifunctional enzyme **glutathione peroxidase-1 (GPX1or GSHC)** was expressed in HIV-infected cells but it was not detected in the counterpart uninfected cells (Figure 2). This enzyme has antioxidant properties and it protects cells (and the body) against the lethal oxidative stress [73]. Elevated levels of GPX1 have been reported in HIV-infected adolescent and young adults with or without antiretroviral drug treatments, both of which cause oxidative stress [74]. In an unrelated study, cells transfected with HIV *env* genes showed a 100% increase in GPX expression [75]. Deficiency or inactivation of GPX1 has been associated with diabetic macrovascular disease, reduced mitochondrial energy production and increased oxidative stress, indicating its protective role *in vivo* [73], [76]. These characteristics may also be critical for maintaining cellular integrity during virus replication (SR unpublished observations). An important property of GPX1 related to lipid metabolism is that the quantity of this protein correlates with the production of malondialdehyde, a major endogenous lipid peroxidation product that has been considered as a marker of oxidative damage (SR unpublished observations) [73], [77]. Overexpression of GPX1 has also been reported to disrupt insulin functions by quenching intracellular reactive oxygen species required for insulin sensitivity [78]. By the use of Ingenuity Systems' bioinformatics programs, GPX1 was found to be significantly associated with enhanced quantity of fatty acids (p = 0.01)(Table 1).

Among the seven *de novo* synthesized enzymes in HIV-infected cells, **protein kinase C, beta type (KPCB or PKC)** showed the highest peak of expression with a significance score of p = 0.02 in relation to the synthesis and metabolism of lipids in humans (Table 1; Figure 2). PKC is a phospholipid-dependent serine-threonine kinase, which regulates the production of phosphatidylethanolamine [17], [79], [80]. Among the many cellular functions associated with the expression of KPCB /PKC, the synthesis and secretion of lipids by epithelial cells appear to be most relevant to our research goals [81]. Overexpression of this kinase has also been shown to cause functional changes that lead to hyperglycemia, lipid-associated diabetic vascular complications, and cardiomyopathy in humans [80], [82]. Upregulation of KPCB /PKC in eosinophils has been linked to the formation of non-nuclear lipid bodies and induction of platelet-activating factor (PAF), which increases the synthesis of eicosanoids (polyunsaturated fatty acids or arachidonic acids) [83]. These data provide overwhelming evidence that activation of PKC enzyme is essential for lipid metabolism [82].

An interesting observation of our study was that the **long-chain-fatty-acid-CoA ligase 1** **(ACSL1: synonym,** **acyl-CoA synthetase** ***)*** was detected within 1.5 hrs after HIV infection, while most other differentially expressed proteins were not quantifiable at this time. This enzyme was induced *de novo* after HIV infection of T-cells *in vitro* and was not detected in any of the numerous gels from the uninfected counterpart cells (Figure 2). The lipogenic activity of this enzyme is also essential for fatty acid metabolism in various tissues as it provides activated intermediates for complex lipid synthesis, protein modification and beta-oxidation [84], [85]. Our statistical and bioinformatics analyses identify ACSL1 to be critical for increased quantities of fatty acids (p = 0.02) and storage of fatty acids as triglycerides (p = 0.005) (Table 1).

The **fatty acid binding protein epidermal (FABP5 or FABPE**) protein was expressed exclusively in the experimentally HIV-infected T-cells (Figure 2; Table 2). Although a recent proteomics study reported FABPE to be slightly downregulated after HIV infection [57], we did not detect this protein in multiple gels of uninfected cells tested at various stages of cell growth and replication. This may be due to differences in the host cells (C33A cervical carcinoma and PML cells) and the virus (HIV-LAI) used in that study [57]. However, in normal physiological conditions, FABP5 is expressed during differentiation of human skin, heart, intestine and adipose tissues [86]. Since FABP5 binds to fatty acids with high specificity, there is a breakdown in the storage of fats during lipolysis, resulting in the release of free fatty acids into the bloodstream [87]. An increase in the expression levels of FABP5 after HIV infection of T-cells *in vitro* suggests its role in producing free fatty acids at a higher rate in these cells (p = 0.02) (Figure 2 &Table 2).

### 3. Overexpression of Low Density Lipoproteins and their Receptors

Our proteomics analyses indicated that the **low-density lipoprotein (LDL) receptor 1 (LRP1)** was excessively upregulated post-HIV infection with 148% higher quantity than detected in the uninfected control cells (Table 2 and Figures 3). This receptor is one of the most important regulators of lipid metabolism and is involved in cell signaling, transportation, and aggregation of lipids through a wide range of mechanisms [88]–[90]. Unlike the high affinity LDL receptors that bind to LDL particles and internalize them for degradation and clearance, LRP1 binds to multiple ligands but more specifically to *aggregated* LDL (AgLDL) [88], [91]. Since LRP1 is the receptor for AgLDL, it plays a significant role in altering signal transduction pathways, and this property distinguishes LRP1 from “classical” LDL receptors [92]. The aggregated AgLDL particles provoke accumulation of cholesteryl esters and modulate adipocyte differentiation-related proteins on the surface of lipid droplets [92]. Interaction of LRPs with cell surface proteoglycans *in vitro* has been shown to induce catabolism of triglyceride-rich lipoproteins [91]. Increased expression of LRP1 is also positively associated with increased quantity of *free* fatty acids [90]. LRP1 is normally associated with macrophages and vascular smooth muscle cells and is upregulated in lipid-enriched plaques in atherosclerotic lesions [88], [92], [93]. In addition, lipid peroxidation of low and very low density lipoproteins has been implicated in early stages of heart diseases through multiple potential pathways [94], [95]. Overexpression of LRP1 in our experimentally HIV infected cells exemplifies its multiple roles in enhancing the quantity of fatty acids (p = 0.02), increasing free fatty acids (p = 0.005), removal of lipids (p = 0.0002) and clearance of triacylglcerol (p = 0.0005) (Table 2).

In addition to LRP1, the HIV infected cells expressed **the Very Low-Density Lipoprotein Receptors (VLDLR**'**s** ***)***. These receptors were not expressed in any of the uninfected cells at different phases of growth (Figure 2 & Table 2). Bioinformatics analysis indicated that the expression of VLDLR's is highly significant in increasing the quantity of fatty acids and triglycerides/free fatty acids (p = 0.01 & p = 0.005 respectively). In addition these receptors are essential for the transport and depletion of lipids (p = 0.0002) and clearance of triacylglycerol (p = 0.0005). The VLDLR's bind to very low density lipoproteins and mediate their catabolism *in vitro* [96]. Thus, expression of both VLDLR's and LRP1 would not only be expected to increase the quantities of LDL and VLDL, but would also reduce clearance rates of very low density lipoproteins into denser particles in both treatment-naïve and anti-retroviral treated individuals [97]–[99]. This is a major cause of lipodystrophy in HIV-infected individuals regardless of treatments [97], [100], [101].

Among all the differentially regulated proteins the **Apolipoprotein-B100 (APOB)** showed the highest quantity with a 1327% increase after HIV infection compared to the minimum detectable levels present in uninfected (control) cells (Figure 3). The APOB is associated with the low density lipoproteins (LDL), intermediate density lipoproteins (IDL), and very low density lipoproteins (VLDL) particles. In both treated and untreated HIV patients, the APOB, VLDL and IDL have been associated with fractional clearance rates [97], [98], [102]. Expression of APOB is clinically significant as it assembles atherogenic lipoproteins involved in plaque formation [103]. Functional bioinformatics analysis indicated that APOB enhances the quantity of both the cell-associated and cell-free fatty acids (p = 0.01 & p = 0.005 respectively). In addition, this protein increases secretion and mobilization of various cholesterols (triacylglycerols) in cooperation with apolipoprotein A1 (p = 0.01). *In vitro* studies have also indicated that the lipidation of APOB results in the conversion of LDL *from* HDL *to* VLDL [104]. An increase in LDL subclass B has also been associated with perturbation of lipid metabolism and dysregulation of triglycerides in patients with AIDS suggesting its role in progression of cholesterol-associated metabolic abnormalities [105].

In HIV-infected cells **apolipoprotein-A1 (APOA1** ***)*** was synthesized *de novo* post-HIV infection and it was not detected in any of the multiple samples tested from the uninfected counterpart cells (Figure 2 & Table 2). Although, APOA1 has been considered as a major protein component associated with high density lipoprotein (HDL) or “good” cholesterol [106], our bioinformatics analyses using Ingenuity's Global Functional Analysis programs indicate that APOA1 is one of the most active proteins that performs multiple functions in lipid metabolism, all of which are statistically significant p-value (p = 0.02) (Figure 1). For example, APOA1 by itself is sufficient for recruitment of cholesterol and sphingomyelin; depletion of phosphatidylcholine; mobilization and translocation of cholesterol; secretion of lysophosphatidylcholine and cholesterols. In cooperation with LRP1, APOA1 helps in the transport of cholesterol esters (p = 0.002). Coordinated expression of APOA1 with C3 (CO3), LRP1, and VLDLR in HIV infected cells is highly significant in the removal of lipids (p = 0.0002). All of these proteins (LRP1, C3 and VLDLR) were co-expressed and excessively upregulated in our experimentally HIV infected cells suggesting their functional interactions (Table 1 and Figure 2). The activated APOA1 also has a proteolytic enzyme activity which is capable of degrading APOB-100 into smaller fragments [107]. Thus, an amphipathic peptide analogue of APOA1 has been reported to inhibit cell fusion in both herpes simplex virus (HSV) and HIV infected cells [108], [109]. Specific subclasses of APOA1 have also been effective in enhancing cholesterol efflux from cells in various phases of reverse cholesterol transport [106].

In an unrelated proteomics-based study, detection of APOA1 in the plasma of HIV-infected individuals was considered a “biomarker for HIV diagnosis” in patients with AIDS. The “diagnostic” significance of APOA1 needs to be further studied as these patients had AIDS and some were on HAART for 2 months [110]. However, it is evident that APOA1 is synthesized *de novo* in the experimentally HIV infected cells without any influence of additional factors. These data indicate that the biological functions of this protein are more versatile than previously envisioned and the multiple roles played by APOA1 in different aspects of lipid metabolism are highly significant (p = 0.0001 to 0.02).

### 4. Lipid Oxidation and Crystallization

An unexpected finding of our proteomics study was that significant amounts of **albumin (ALBU)** precursor protein were synthesized *de novo* in HIV-infected cells i.e. not detected in any of the uninfected counterpart cells in wide range of growth phases over a period of >3 months (Figure 2). Albumin is a major multifunctional secretory/transporter protein that binds to hormones, bilirubin, drugs, a variety of elements Ca (2+), Na (+), K (+) and other molecules present in the plasma [111]. The human ALBU also binds to fatty acids with high affinity as it has several polymorphic forms with long hydrophobic pockets that are distributed asymmetrically throughout the repeating domains of this protein [111], [112]. The binding of ALBU to fatty acids regulates enzymes that metabolize, esterify and crystallize lipids [113]. The upregulation of ALBU, a “normal” protein is therefore highly significant (p = 0.02) as it plays a significant role in crystallization of cholesterol. The high affinity binding of fatty acids to ALBU *in vivo*, also results in excessive secretion of APOB, leaving fatty acids unavailable for lipoprotein assembly in hepatocytes [114]. The HIV-infected individuals who show higher LDL levels in the plasma also have albuminaemia indicating coordinated upregulation of ALBU with increased lipid profiles [5]. In the present study expression of lipogenic proteins correlated with the expression of ALBU in HIV-infected cells (Figure 2 & Table 2). Another most relevant finding related to our experimental results was that binding of ALBU to the complement C3 protein results in the formation of microbubbles that adhere to vascular endothelium and accelerate atherosclerotic processes [115]. As noted earlier, both ALBU and the C3 proteins were expressed only in HIV-infected cells and these proteins were not detected in uninfected cells, suggesting their putative co-operative roles in inducing lipid abnormalities (Figure 2).

### 5. Effects of Downregulated Proteins on Lipid Metabolism

The differential proteomics analyses of HIV-infected and uninfected cells identified four proteins (**ACBP, PDIA3, STIP1 & LRP2**) that were downregulated post-HIV-infection (Figure 3). Functional bioinformatics analyses indicated that suppression of these proteins could be associated with increased production of proteins that enhance lipid synthesis and quantity (Figure 1). For example, **Acyl-CoA binding protein** **(ACBP** ***)*** is a key protein that modulates the long- chain fatty acyl-coenzyme-A (Acyl-CoA) metabolism [116]. This protein is normally induced during adipocyte differentiation and its downregulation inhibits adipocyte differentiation which results in growth arrest and adipocyte cell death [117]. The slight decrease in the expression of ACBP in HIV infected cells (Figure 3) suggests that downregulation of this protein may be potentially harmful for adipocyte differentiation *in vivo*. ACBP also facilitates esterification of long-chain fatty acids with coenzyme (Acyl-CoA) which is the first step in fatty acid metabolism [116]. Depletion of this protein in cells has been shown to result in abnormalities of lipid metabolism, particularly those involved in fatty acyl-CoA long chain formation [116], [118]. Loss of ACBP also leads to triacylglycerol biosynthesis abnormalities as it binds long-chain fatty acyl-CoA esters and facilitates its transportation [118], [119]. The downregulation of ACBP therefore significantly enhances abnormalities and mobilization of triglycerides (p = 0.02).

The quantity of **protein disulphide isomerase (PDIA3),** previously known as **phosphoinositol-specific phospholipase C** was about 9% lower in HIV-infected cells than the uninfected cells (i.e.downregulated) (Figure 3). Although in another study, PDIA3A was shown to be upregulated 2.43-fold after 42 hours of “massive” (high multiplicity of HIV infection), this may be due to variations in the HIV strain (LAI clone) used for this study, the macrophage tropism of the virus, the cells used to grow the virus ( C33A human cervical carcinoma monolayer cells and PM1 T cells) [57], [120]. While the expresssion of PDIA3A was initially reported to be important in the reproductive tract and placenta [121], this peptidase is shown to be critical for disulfide bond formation of newly synthesized proteins [119], [122]. PDIA3 also plays an important role in signal transduction from the cell surface receptor to other parts of the cells including the nucleus and endoplasmic reticulum [121]. This protein is identical to the beta subunit of the microsomal triacylglycerol transfer protein (MTP), which is a multifunctional protein essential for the assembly, folding, and secretion of APOB-containing lipoproteins [119], [122], [123]. The absence or downregulation of the beta subunit may cause defective secretion of APOB-containing lipoproteins, which would then promote development of beta-lipoproteinemia, an autosomal recessive disease [122], [123]. During lipid metabolism, PDIA3A is also significantly associated with the cleavage of phosphatidylinositol (p = 0.02).

The **stress-induced-phosphoprotein 1 (STIP1** ***)*** was downregulated post-HIV infection (Figure 3). This is a co-chaperone or an adapter for the human **heat shock proteins (HSPs)** **Hsp70/Hsp90 (or HS7H and HS9B)** machinery which organizes complex interactions with other proteins [124], [125]. Both HS7H and HS9B were *upregulated* exclusively in HIV-infected T-cells and were not expressed in uninfected cells (Figure 2; Table 2). In general these proteins are expressed in response to the endogenous stress and they protect cells from injury (i.e. inhibit apoptosis) [126], [127]. In physiologic conditions, coordinated expression of HS7H and HS9B stabilizes preexisting proteins against aggregate formation and they mediate correct folding and maturation of newly synthesized cellular proteins [126], [128], [129]. During the assembly of multicomponent complexes, HS7H and HS9B alter conformations of substrate proteins as and when needed for specific signal transductions [130]. HS7H and HS9B are hormone binding proteins and proper assembly of their complexes with progesterone and glucocorticoid receptors depend on the transcriptional activation subsequent to hormone binding [131]–[134]. In an experimental model both progestin and androgen were shown to enhance production of triglycerides particularly after the induction of fatty acid synthetase in cells [135].

An important and pertinent function relevant to our experimental model is that Hsp70 binds to the activated protein kinase C (PKC) and prolongs the life of the mature kinase by allowing the enzyme to re-phosphorylate [126], [136]. As discussed earlier, PKC (KPCB) was overexpressed in HIV infected cells (Figure 2) and it is an essential enzyme for the generation and regulation of phosphatidylethanolamine that activates diacylglycerol [82], [126], [136]. Any disruption of this interaction would prevent re-phosphorylation of PKC which is essential for multiple functions including fatty acid synthesis. It is therefore not surprising that both HS7H /HS9B complex and PKC were induced *de novo* after HIV infection. The relationship of these proteins to lipid metabolism was scored to be highly significant (p = 0.0002).

The **LDL receptor-related protein 2 (LRP2)** was downregulated in HIV-infected cells 3 hrs post- infection (Figure 3 & Table 2). This is a multi-ligand receptor (megalin) and it participates in a wide range of physiological functions, including protection against atherosclerosis [89]. Downregulation of LRP2 in HIV-infected cells with concomitant upregulation of LRP1 suggests an enhanced production of lipid aggregates and related abnormalities.

### 6. Statistical Significance of Protein Interaction Pathways

All biological processes are regulated by interactions of proteins with other proteins, lipids, lipoproteins, RNA or DNA in order to perform specific cellular functions. Although a complete network of all possible protein-protein interactions in the cell is difficult to visualize, we have constructed protein-interaction pathways of 18 proteins that were identified to be significantly associated with different aspects of lipid metabolism (p-values ranging from 0.0002 to 0.01;Tables 1 and 2).

**Figure 4:**
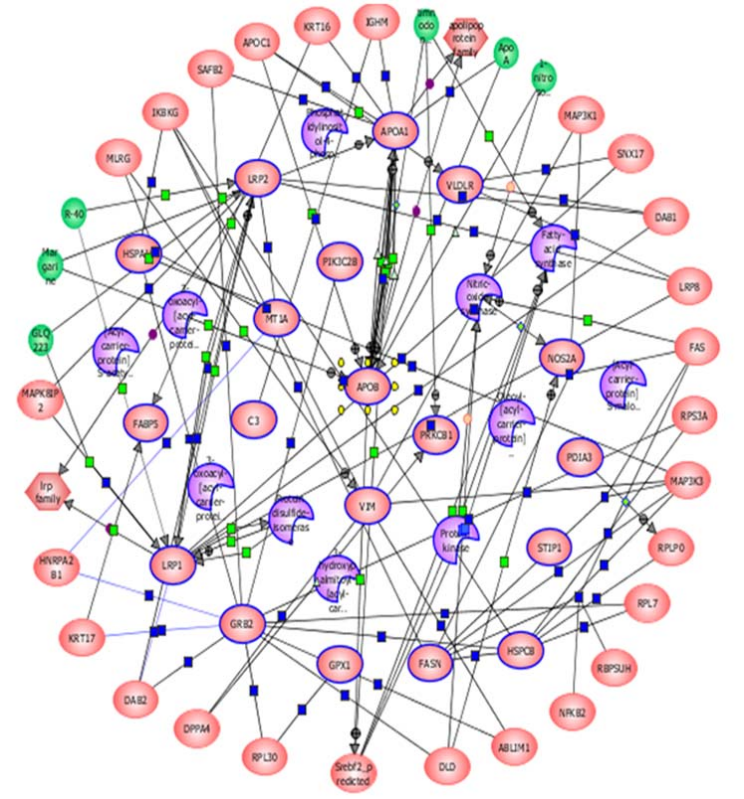
Protein-interaction pathways of proteins associated with various aspects of lipid metabolism. The functional significance of each protein was defined using Ingenuity bioinformatics platforms designed for Systems Analyses of genes and protein expressed in human health and disease. All proteins were uploaded into the Strategene Pathway Architect (SPA) program and protein-interaction pathways were constructed according to the manufacturer's instructions. Proteins identified by our present proteomics studies are shown with blue outlines; purple-colored proteins are enzymes and kinases and pink-colored (with blue outlines) are other significant proteins involved in the lipid metabolism. Lines connecting proteins indicate molecular networks of interactions. The greater the number of lines originating from a protein, the more significant the molecular interactions of that protein with other protein/s. Note the extensive networks of interactions between all proteins identified in the present study (blue circles), particularly those of APOB and APOA-1. Full protein names, abbreviations and accession numbers of each protein are listed in Tables 1&2.

Two different programs were used to construct interaction pathways (Ingenuity Pathway Analysis (IPA) Systems and Strategene Pathway Architect (SPA) software). Although both programs generated conceptually similar pathways, the interaction diagrams presented here were created by Strategene Pathway Architect according to the instructions provided by the manufacturers (Figure 4).

Using the IPA's bioinformatics programs which integrate the physical, biochemical and biological properties of proteins, we have created tentative scenarios for the 18 proteins whose expression or suppression has been significantly associated with putative steps or functions involved in lipid metabolism (p = <0.0002–0.01) (Figure 1). Among the 18 differentially regulated proteins produced in response to HIV infection, FAS, FABPE, KPCB, and P3C2B enzymes/kinases have been shown to be essential for the synthesis and production of fatty acids; the multifunctional molecular chaperon machinery (HS71L/ HS90B) protects PKC kinase and prolongs the life of this enzyme in the cell while preventing other newly synthesized proteins from incorrect folding [126]. Binding of HS71L/ HS90B proteins to progesterone receptors generates signals for the production of triglyceride in the cell [135]. The upregulation of APOB, CO3, LRP1, VLDLR, GPX1, and ACSL1 and slight downregulation of LRP2 have been associated with increased quantities of free fatty acids; the overproduction of APOB and *de novo* synthesis of APOA1 are associated with higher lipid secretion; upregulation and concomitant expression of APOA1, LRP1, VLDLR, and CO3/C3 is critical for lipid depletion, clearance and transport; APOB, together with slightly downregulated ACBP, mobilizes the fatty acids and produces triacylglycerol abnormalities. Production of ALBU esterifies, crystallizes and produces aggregated or oxidized lipids /cholesterols.

An increased production of APOB-100, VLDLR, and LRP1 in HIV-infected cells also indicates that multiple lipid metabolic pathways are triggered by HIV replication in T-cells. These interactions may induce physiological changes related to lipid metabolism and cholesterol homeostasis, as well as formation of aggregated lipids that bind to LRP1 (Figures 1 & 4). As the production of lipid- inducing proteins increases, the numbers of receptors for these ligands are also augmented and new signal transduction molecules are activated. This cascade of events would result in a breakdown of multiple metabolic pathways, especially in HIV-infected individuals.

### 7. Antiretroviral Drug does not affect Production of Apolipoproteins

**Figure 5:**
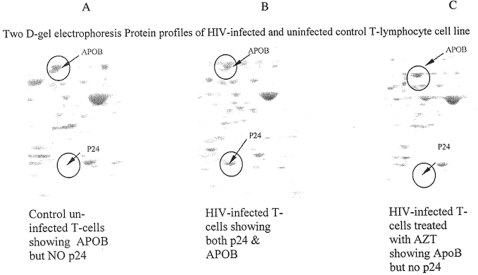
Figure 5: Two-dimensional gel electrophoresis patterns of HIV-infected and counterpart uninfected cells T-cells. Protein profiles were evaluated 48 hours after treatment with azidothymidine (AZT), or mock treatment with phosphate buffered saline. Figure 5A represents protein profile of uninfected T-cells and shows theexpression of Apolipoprotein B-100 (APOB) and the absence of HIV p24 antigen. Figure 5B represents the protein profile of HIV-infected T-cells and displays the presence of both APOB and p24 antigen. Figure 5C indicates that APOB is NOT inhibited by treatment of HIV-infected cells by AZT while the viral proteins are inhibited by the antiviral drug.

One of the most significant proteins associated with many lipid related diseases is APOB-100 (p = 0.01 to 0.005). As indicated earlier, this protein was excessively upregulated in HIV-infected cells post HIV-infection (Figure 3). Presence of APOB promotes cholesterol efflux and it is absolutely essential for the metabolism of low-density lipoproteins (LDL), the intermediate density lipoproteins (IDL) and the very low-density lipoproteins (VLDL). To validate the sensitivity and specificity of our proteomics technology and the functionality of both viral and cellular proteins, we analyzed the expression of the critical APOB protein in HIV infected, uninfected and counterpart cells treated with an inhibitor and antiviral drug azidothymidine (AZT or Zidovudine). This drug has been shown to inhibit HIV replication *in vitro* and is used in combination with other drugs to treat HIV patients [137], [138]. As can be seen in the three sequential gel images of Figure 5, APOB is NOT inhibited by treatment of HIV-infected cells by Zidovudine while HIV p24, the major gag protein is inhibited. As expected, the HIV-infected cells expressed P24 and the uninfected cells did not. These results not only validate our techniques but also indicate that cellular pathways for the synthesis of APOB are distinct from those of HIV replication (Figures 4 & 5).

### 8. Conclusions

We conclude that the replication of HIV alone in human T- cells modulates synthesis of novel enzymes, kinases and other proteins that enhance fatty acid synthesis, increase lipid peroxidation (crystallization), disrupt lipid metabolism and reduce lipid clearance. Since HIV-modulated proteins were isolated by a rapid extraction technique and fractionated without the use of any affinity purification techniques such as protein-specific ligands or antibodies, we believe this unbiased proteomics approach has led to the discovery of novel proteins that have not been identified previously in HIV-infected cells.

Our data provides direct evidence that HIV infection alone can induce a number of novel lipid-associated proteins without any influence of genetic or epigenetic factors that influence metabolic events *in vivo*. Although APOB and VLDL, IDL have been associated with fractional lipid clearance rates in HIV-infected individuals [97], [98], [102], expression of proteins involved in different aspects of lipid metabolism has not been reported. We have validated our techniques and demonstrated that treatment of HIV-infected cells with antiviral drug Zidovudine does not inhibit the production of APOB, one of the most critical lipid associated proteins while the HIV replication is inhibited (Figure 5). APOB is a secretory globin-like protein (lipoprotein ) responsible for carrying LDL, which supplies cholesterol to the tissues. Excess lipids are transported through the blood by lipoproteins and are stored in adipocytes. In HIV-infected individuals both protease inhibitors and nucleoside reverse transcriptase inhibitors such as AZT (Zidovudine) have been shown to have detrimental effects on adipose tissue, liver, and muscle [6], [7], [18], [22].

Bioinformatics and protein- interaction pathway analyses of the 18 differentially regulated proteins indicated that these proteins are biologically relevant as many have been reported in non-HIV infected patients with hyperlipidemia and other lipid-related disorders. *In vivo* the chronically HIV-infected cells produce many secretory cytokines/factors that bind to adipocytes and other cell types (bone marrow, connective tissue, mesenchymal stem cells and others) resulting in enhanced signaling for the production of fatty acids and lipid-regulatory enzymes. Since lipids themselves are excellent signal- transduction molecules, they can bind to cells nonspecifically and induce new signals in both infected and uninfected cells. A cascade of events would then follow before the lipid-related diseases are apparent *in vivo*.

Translational studies using knockout mice and characterization of the newly discovered proteins by testing the effects of small interfering RNA (RNAi) or small- molecule inhibitors *in vitro* would help in identification of most prevalent, functionally relevant and statistically significant biomarkers for early diagnosis of individuals who might be at high risk for developing lipid-related disorders. Future studies may lead to innovative strategies for developing better therapeutics or inhibitors for preventing lipid-related metabolic disorders.

This is the first study to experimentally demonstrate correlation between HIV replication and *de novo* expression and/or upregulation of proteins that enhance production of key proteins involved in lipid metabolism.

## Materials and Methods

The primary goal of this study was to apply proteomics-based approaches to study HIV-modulated protein profiles overtime under controlled conditions *in vitro.* The study circumvented problems associated with numerous environmental and genetic factors that influence HIV infection, lipid production and disease progression *in vivo*.

### Cell Cultures and HIV Infection

To address some of the basic questions related to the virus-host cell interactions, we conducted several labor-intensive proteomics studies to identify proteins modulated by HIV infection alone. Each step of these studies, from sample preparation to data analyses, has been quality- controlled in order to understand the complexities of HIV replication. A T-cell line (RH9) was chosen because HIV replication in these cells is highly efficient and both the HIV and the cell are clonal derivatives. The genome-wide protein profiles solely in response to HIV infection of cells *in vitro* were evaluated without any additional supplements or factors that may possibly influence lipid functions. Cells were grown in RPMI medium containing 10% fetal bovine serum and 2mM glutamine with no antibiotics or any cytokine [58].

Approximately 6–7×10^8^ cells were suspended at a concentration of 2×10^6^ cells/ml of medium containing 2 ug/ml polybrene. After 24 hrs, one aliquot of 2×10^7^ cells was removed as a “baseline control” and remainder of cells were divided equally into two large, 125 cm flasks. One set of cultures was infected *in vitro* with a biological clone of HIV-1(X4 strain) at a multiplicity of infection of one (MOI = 1) while the counterpart uninfected cells were maintained as the experimental control. Cultures were incubated at 37°C in a humidified atmosphere of 5% CO2. After 90 minutes, both sets of cultures were transferred to centrifuge tubes to spin down cells and to remove the medium and /or the virus. All cells were washed once with fresh medium and transferred to new flasks. At this time (i.e. after 1.5 hours), an aliquot of 2×10^7^ cells from each culture was removed for analysis and the rest of the cells were incubated as before. Subsequently, both the HIV-infected and uninfected (control) cell samples were collected at multiple time points up to 96 days (i.e. different stages of infection and cell growth at 3 hr, 6 hr, 12 hr, 24 hr, 48 hr, 72 hr, 96 hr, 6 days (d), 10 d, 15 d, 26 d, 47 d and 96 d post-infection . In addition, we tested at least ten different infected and uninfected sets of cells between 10–26 days of infection and two separate aliquots grown over a 3 month period from an uncloned culture of the same cell line (H9) that was infected with the X4 HIV strain in 1990's. This culture has been maintained in our laboratory *in vitro* as a chronically infected, virus-productive cell line.

Supernatants of all cell cultures were harvested for protein analyses, regardless of their time of collection and were tested for the presence of HIV P24 antigen (Abbott's HIV-p24 antigen test).

### Two- Dimensional Gel Electrophoresis (2DGE) and Image Analysis

Proteins were extracted from approximately 2×10^7^ cells from each of the infected and uninfected sets of cultures at each time point using differential extraction procedures established in our lab [139]. Cell pellets were briefly washed twice for 5 minutes each in the phosphate buffered saline (PBS) to remove serum and other pertinacious material, followed by a second wash in Tris (hydroxymethyl)aminomethane. Cells were then solubilized by a sequential two-step procedure using two separate reagent concentrations (5–8 M urea, 2–4% CHAPS, 2 M thiourea and other nonionic and zwitterionic detergents with 2.5%carrier ampholytes and protease inhibitor cocktail (BioRad)). The solubilization steps were modified by a rapid lysis technique (10–15 seconds followed by 2 seconds' sonication) according to our laboratory's standardized protocols [139]. The first fraction contained the most soluble membrane proteins followed by other membrane, cytoplasmic, and nuclear protein fractions [139]. Each sample was analyzed by isoelectric focusing (IEF), two-dimensional gel electrophoresis (2DGE), image analysis, and Matrix Assisted Laser Desorption Time Of Flight (MALDI- TOF) mass spectrometry as described below.

For IEF, protein fractions were clarified by centrifugation at 100,000×g, and the supernatant was analyzed in the first dimension on a pH gradient of 3–10. Each IEF strip containing separated proteins was then analyzed by 2-dimensional gel electrophoresis (2-DGE) on 6–18% gradients of SDS-polyacrylamide gels. To analyze low abundant proteins, cell lysates were separated by IEF on different overlapping pH gradients (3–10, 4–7 and 5–8) and then size fractionated by molecular weight using 2DGE on different gradient gels. Each of the HIV-infected and uninfected counterpart samples collected overtime were processed and run at the same time under identical conditions using a 12-gel electrophoresis tank. For quality control, a gel from a lyophilized-reconstituted sample of E. coli proteins was used as a general standard, and one each of the best gels from HIV-infected and counterpart uninfected samples was used as experimental (i.e.HIV-infected and uninfected cells) reference standard with each run.

Proteins were visualized by staining with Coomassie Blue. Some gels containing proteins of interest were also counterstained with Amido or silver black *after* scanning the images of the Coomassie-stained gels. However, since silver black was not compatible with MS, we used Sypro Ruby red which gave distinctive signals for proteins that were present in both gels but displayed different expression levels. Digitized images of each gel were obtained by CCD camera and protein spots in each of the uninfected and infected samples were analyzed by the use of Melanie and PDQuest softwares. Identical parameters were used for all gel scans including those of the standards and controls against which each image was aligned and matched automatically. Each spot in the uninfected controls was compared with the corresponding spot in gels from the counterpart HIV-infected cells. The software was designed to correct differences in the gel staining according to the algorithms that integrate the optical densities (OD) of each pixel in the spot area and then evaluated normalized quantities against all standardized spot images. Spot intensities were set to differ in the normalized quantities by a factor of 2 compared to the relative average of one group to that of the other. In addition, each gel was critically examined *manually*, and spots matches were corrected if necessary.

Expression or suppression of many differentially expressed proteins was confirmed in separate experiments carried out over a period of approximately 3 years; one experiment was conducted with time point analyses (1.5 hours to 96 days) over a period of 3 months and in subsequent experiments samples were harvested on specific time points ranging from 10–26 days. Although many differentially regulated proteins were detected as early as 1.5 hrs post infection and at several successive time points, most proteins in 1.5 to 4 hrs' gels were not quantifiable due to small quantities. For detailed analyses, we selected 33 gels from a large number of gels run on different IEF (pH 3–10 & 4–7) and SDS -PAGE gradients. These included at different time points ranging from 1.5 hours to 96 days plus numerous samples from 10–26 days as these samples yielded higher quantities and were detected in multiple gels. As indicated earlier, we also used chronically HIV-infected cells and control uninfected cells as standard references for all gels.

All proteins from HIV-infected cells were compared with those expressed in the uninfected counterpart cells at different time points. *Only* those proteins that were reproducibly detected in multiple gels were selected for bioinformatics and statistical analyses. To obtain a more accurate dataset for each of the upregulated or downregulated protein in HIV-infected cells we calculated standard deviations of the mean values from the range of *normalized* quantities expressed in both HIV-infected and uninfected samples tested. A plus and minus one standard deviation of the mean value is illustrated by a bar within the graph for each protein (Figures 2 and 3).

### MALDI-TOF Mass Spectrometry (MS)

All differentially regulated spots (i.e. upregulated, downregulated or *de novo* synthesized proteins post-HIV infection) and some common spots present both in the infected and uninfected cells were selected for protein identification by MALDI-TOF MS. All spots were excised form gels and washed successively with water, ammonium bicarbonate (25 mM, 1∶1(v/v) acetylnitrite (ACN):25 mM ammonium bicarbomnate), and 100% ACN. The gels were then vacuum-dried and stored in the refrigerator until MS was performed. Prior to MS the dried gels were reconstituted in 25 mM ammonium bicarbonate containing 0.1 mg ultra-pure, MS-grade trypsin (pH 7.8) (Promega) and incubated overnight to extract peptides. Peptide fingerprints of in-gel digests were analyzed by MALDI-TOF-MS according to the manufacturer's protocols (Applied Biosystems). All annotated spectra were analyzed using PS1 software (Applied Biosystems) and MASCOT (Matrix Science, London). Embedded in these programs are biostatistical and analytical systems to select differentially expressed peaks (MS signals). The search parameters included mammals and human. All spectra were linearly calibrated using a two-point calibration with internal standards corresponding to autoproteolytic tryptic peaks. To quantify the differences in calibration between the reference spots and experimental protein spots on the plate, we applied the derived ‘topological map’ to afford the best calibration throughout the plate while using a single calibrant.

### Validation of Protein Functionality by the use of an HIV-Inhibitor

To validate the functionality of both viral and cellular proteins expressed in HIV-infected cells, we tested the same H9 T- cells described above and infected these with the same X4 HIV strain that was used for all proteomics studies. Cells were divided into two aliquots; one was infected with HIV and the other was used as uninfected control. The virus in the supernatant was removed after 3 days and cells were placed in new flask after washing with PBS. Each set of cultures was then divided into 4 flasks each (i. e. 8 flasks); two each (i.e. duplicates) of the HIV-infected and uninfected (control) cultures were maintained as experimental controls and separate replicates of each were treated with10 ug/ml of Azidothymidine (AZT or Zidovudine) a reverse transcriptase inhibitor (gift from GlaxoSmithKline/Burroughs Wellcome). This drug has been shown to inhibit HIV replication *in vitro* and has been used to treat HIV infected individuals alone and in combination with other drugs [137], [138]. After 48 hrs, proteins were extracted from all cultures as described above and all samples from the treated, untreated, HIV- infected and uninfected counterpart cultures were tested by proteomics analyses as described above.

The ability of HIV to replicate or to be inhibited by AZT was tested by the presence or absence of p24 antigen in the supernatant and in the cellular protein fractions by proteomics technology (Figure 5). No HIV p24 was detected in HIV-infected cells treated with AZT whereas HIV-infected untreated cells produced significant quantities of this protein indicating that HIV replication was inhibited and the proteomics results are significant. Further, since the expression of APOB protein was not affected by the antiviral drug treatment it indicated that both low abundant p24 and high abundant proteins can be detected by our proteomics technology.

### Statistical Analyses and Protein- Interaction pathways involved in Lipid Metabolism

The significance of proteins involved in lipid metabolism was possible only by analyzing massive amounts of data on HIV-modulated cellular proteomes overtime for a very long period. Several hundred differentially expressed proteins (upregulated or downregulated or *de novo* synthesized proteins post-HIV infection) were identified by proteomics analyses and each was categorized according to the known function/s in the global databases of human proteins (UniProt/SwissProt). Standard deviations of mean values were calculated for each protein quantity and are represented by bars within the graph (Figure 2 & 3). By the use of bioinformatics and computational program “Functional Repository of Humans Genes and Proteins” (Ingenuity Systems Inc.), biological functions of each protein were validated in relation to the proteins involved in lipid synthesis, production, quantity, cleavage, transport, secretion, and/or lipid oxidation. These programs also calculated the likelihood of whether a particular function in “Global Functional Analysis and Global Canonical Pathways” was due to a random chance or a set of proteins identified experimentally could be statistically associated with specific biological processes involved in lipid metabolism.

Using 2×2 contingency tables, the p-values of all experimentally identified proteins were calculated by the Global Functional Analysis programs in relation to the total number of proteins that have been shown to be associated with human lipid metabolism. The p-values of proteins involved in the lipid metabolism were calculated by using the right-tailed Fisher Exact Test and Ingenuity Pathway Analysis Systems. These analyses identified a coordinated expression of 18 proteins with statistical significance (*p* values  = <0.05 as indicators) in relation to global and local lipid metabolism-related functions**.** However, All proteins selected for this study had p-values <0.02.

For protein-interaction pathway analyses, we have used the Ingenuity Pathway Analysis (IPA) System and Strategene Pathway Architect (SPA) software. All 18 proteins involved in the lipid metabolism were used to construct the pathways according to manufacturer's instructions. One of the pathways made by the SPA is presented in Figures 4.
